# A diagnostic window for the treatment of acute graft-versus-host disease prior to visible clinical symptoms in a murine model

**DOI:** 10.1186/1741-7015-11-134

**Published:** 2013-05-21

**Authors:** Carina A Bäuerlein, Simone S Riedel, Jeanette Baker, Christian Brede, Ana-Laura Jordán Garrote, Martin Chopra, Miriam Ritz, Georg F Beilhack, Stephan Schulz, Robert Zeiser, Paul G Schlegel, Hermann Einsele, Robert S Negrin, Andreas Beilhack

**Affiliations:** 1 Department of Medicine II, Würzburg University Clinics, Zinklesweg 10, Würzburg, D-97078, Germany; 2 Interdisciplinary Center for Clinical Research (IZKF), Würzburg, Germany; 3 Graduate School of Life Sciences, Würzburg, Germany; 4 Division of Blood and Marrow Transplantation, Department of Medicine, Stanford University, Stanford, CA, USA; 5 Department of Medicine III, Vienna Medical University, Vienna, Austria; 6 Institute of Pathology Charité, Berlin, Germany; 7 Department of Hematology and Oncology, Freiburg University Medical Center, Freiburg, Germany; 8 Department of Pediatrics, Würzburg University Hospital, Würzburg, Germany

**Keywords:** Allogeneic stem cell transplantation, Graft-versus-host disease, Minor histocompatibility antigen mismatch transplantation

## Abstract

**Background:**

Acute graft-versus-host disease (aGVHD) poses a major limitation for broader therapeutic application of allogeneic hematopoietic cell transplantation (allo-HCT). Early diagnosis of aGVHD remains difficult and is based on clinical symptoms and histopathological evaluation of tissue biopsies. Thus, current aGVHD diagnosis is limited to patients with established disease manifestation. Therefore, for improved disease prevention it is important to develop predictive assays to identify patients at risk of developing aGVHD. Here we address whether insights into the timing of the aGVHD initiation and effector phases could allow for the detection of migrating alloreactive T cells before clinical aGVHD onset to permit for efficient therapeutic intervention.

**Methods:**

Murine major histocompatibility complex (MHC) mismatched and minor histocompatibility antigen (miHAg) mismatched allo-HCT models were employed to assess the spatiotemporal distribution of donor T cells with flow cytometry and *in vivo* bioluminescence imaging (BLI). Daily flow cytometry analysis of peripheral blood mononuclear cells allowed us to identify migrating alloreactive T cells based on homing receptor expression profiles.

**Results:**

We identified a time period of 2 weeks of massive alloreactive donor T cell migration in the blood after miHAg mismatch allo-HCT before clinical aGVHD symptoms appeared. Alloreactive T cells upregulated α4β7 integrin and P-selectin ligand during this migration phase. Consequently, targeted preemptive treatment with rapamycin, starting at the earliest detection time of alloreactive donor T cells in the peripheral blood, prevented lethal aGVHD.

**Conclusions:**

Based on this data we propose a critical time frame prior to the onset of aGVHD symptoms to identify alloreactive T cells in the peripheral blood for timely and effective therapeutic intervention.

## Background

Allogeneic hematopoietic cell transplantation (allo-HCT) is the only curative treatment option for many malignant diseases due to the beneficial immunological graft-versus-leukemia (GVL) effect [1]. However, (acute) graft-versus-host disease ((a)GVHD) continues to be a major complication after allo-HCT. This syndrome is caused by donor T cells that attack the intestinal tract, liver, and skin [2]. Early diagnosis remains challenging and to date mainly relies on clinical symptoms and histopathology. When clinical symptoms are readily detected, response to therapy is rarely beneficial to the patient. Therefore, a predictive test to identify patients at risk before the visible onset of aGVHD is highly desirable.

A detailed understanding of the pathophysiological processes including the kinetics of alloreactive T cell priming, migration, and effector mechanisms after allo-HCT may provide clues to identify patients that are likely to develop aGVHD. Recently, *in vivo* bioluminescence imaging (BLI) of T cells that carried the firefly luciferase (*luc+*) gene in a major histocompatibility complex (MHC) mismatched mouse model helped define a distinct aGVHD initiation and effector phase [3]. Donor T cells first migrated into secondary lymphoid organs (SLOs), where they were activated by interacting with host antigen presenting cells (APCs). This interaction led to the upregulation of certain homing receptors which the cells used for target tissue infiltration [3]. The subsequent effector phase, taking place in peripheral aGVHD target tissues, started 3 days later when alloreactive T cells left the SLOs and migrated via the peripheral blood (PB) to their respective target organs, mainly the gastrointestinal tract (GIT), the liver, and the skin [4-6].

In the present work, we asked whether it is possible to detect alloreactive donor T cells in the PB early after allo-HCT in a clinically relevant minor histocompatibility antigen (miHAg) mismatch mouse model. We characterized donor T cells according to the expression of two well-described homing receptors, α4β7 integrin and P-selectin ligand. Based on these observations we succeeded in effectively treating mice with rapamycin on first alloreactive cell detection in the PB to timely prevent aGVHD exacerbation.

## Methods

### Ethics statement

All experiments were performed according to the German regulations for animal experimentation and approved by the Regierung von Unterfranken as the responsible authority (Permit Number 55.2-2531.01-30/09).

### Mice

All HCT experiments were performed with sex-matched 8 to 12-week-old mice. BALB/c (H-2^d^, CD90.2) and C57Bl/6 (H-2^b^, CD90.2) mice obtained from Charles River (Sulzfeld, Germany); BALB/b (H-2^b^, CD90.2) and congenic C57Bl/6 (H-2^b^, CD45.1) obtained from Jackson Laboratories (Bar Harbor, ME, USA). The luciferase-expressing (*luc*+) transgenic FVB-L2G85 line [7] was backcrossed over 12 generations onto C57Bl/6 (H-2^b^, CD90.1) background. Mice were housed in a pathogen-free facility at the Center for Experimental Molecular Medicine (ZEMM), Würzburg, Germany.

### Flow cytometry analysis

Cells were stained with the following antibodies (clones): anti-CD8α (53-6.7), anti-CD4 (RM4-5), anti-CD90.1 (H1S51), anti-CD45.1 (A20), anti-LPAM-1/α4β7 (DATK32) from Biolegend (Uithoorn, The Netherlands) and eBioscience (Frankfurt, Germany), P-selectin ligand-IgG fusion protein (Becton Dickinson (BD), Heidelberg, Germany), anti-human-IgGκ-FITC (Jackson ImmunoResearch Laboratories, West Grove, PA, USA). Dead cells were excluded with propidium iodide (PI) or 4′,6-diamidino-2-phenylindole (DAPI) staining. Flow cytometry was performed on a FACS-Canto II (Becton Dickinson), and data was analyzed with FlowJo Software version 8 (Treestar, Ashland, OR, USA). Gates were set using the fluorescence minus one-gating strategy [8]. Anti-mouse or anti-rat/hamster CompBeads (BD) were used for compensation controls.

### HCT

Recipient mice (BALB/c, BALB/b, and C57Bl/6) were myeloablatively irradiated (BALB/c, BALB/b: 8 Gy; C57Bl/6: 9 Gy) with an electron linear accelerator (Mevatron Primus, Siemens, Germany). Bone marrow (BM) cells from wild-type (WT) C57Bl/6 donor mice were flushed from femur and tibia bones with phosphate-buffered saline (PBS; PAN, Aidenbach, Germany). T cells were isolated from spleens of *luc*^*+*^ C57Bl/6-L2G85 donor mice and red blood cells were lysed. Splenic CD3^+^ single cell suspensions were enriched using the Dynal Mouse T cell Negative Isolation Kit (Invitrogen, Darmstadt, Germany) according to the manufacturer’s protocol. Cell purity of the CD3^+^ was confirmed by post-enrichment fluorescence-activated cell sorting (FACS) analysis (>90%) For hematopoietic reconstitution, all recipient mice were injected intravenously with 5 × 10^6^ C57Bl/6 WT BM cells. To induce aGVHD, BALB/c recipients (MHC major mismatch model) and syngeneic C57Bl/6 recipients were coinjected intravenously with 1.2 × 10^6^ enriched CD3^+^/*luc*^*+*^ and 5 × 10^6^ BM cells, while BALB/b recipients (miHAg mismatch model) were coinjected intravenously with 5 × 10^6^ CD3^+^/*luc*^*+*^ T cells and 5 × 10^6^ BM cells within 3 h after irradiation. Transplanted mice were monitored daily for survival, weight change, and clinical GVHD symptoms according to Cooke *et al*. [9].

### Immunosuppressive treatments

For *in vivo* studies, rapamycin (Wyeth, Reading, UK) was dissolved in carboxymethylcellulose sodium salt (C-5013; Sigma-Aldrich, Munich, Germany) and polysorbate 80 (P-8074, Sigma-Aldrich) to a final concentration of 1.5 mg/kg body weight (BW) [10] and injected intraperitoneally daily from day +6 to day +15 in a final volume of 100 μL.

### *In vivo* bioluminescence imaging

Mice were anesthetized and coinjected intraperitoneally with 80 mg/kg BW ketamine (Pfizer, Berlin, Germany) and 16 mg/kg BW xylazine (CP Pharma, Burgdorf, Germany) together with d-luciferin (Biosynth AG, Staad, Switzerland) at a dose of 150 μg/g BW. Three or six mice per group per day were imaged for analysis. Images were captured as previously described [5,7] using an IVIS Spectrum charge-coupled device (CCD) imaging system (Caliper-Xenogen, Alameda, CA, USA). Imaging data were analyzed and quantified with Living Image Software 3.1 (Caliper-Xenogen).

### Peripheral blood samples

Mice were bled daily (three mice per group) via the tail vein and erythrocytes were lysed for FACS analysis. In addition, 25 μL PB from each mouse was added to 100 μL PBS/EDTA (1 mM) for white blood cell counts using a Sysmex XT-2000i (Horgen, Switzerland).

### Immunofluorescence staining and immunohistochemistry

Organs were embedded in optimal cutting temperature (OCT) compound (Sakura, Zoeterwoude, The Netherlands) and cut into 5-μm thick sections. Slides were kept at -20°C until staining. After air drying and acetone fixation (10 minutes at room temperature), sections were incubated with blocking solution for 15 minutes (PBS + 1% fetal calf serum (FCS)) prior to staining with the following antibodies for 1 h: CD8α-Alexa488 (clone 53-6.7, Biolegend), CD90.1-APC (clone HIS51, eBioscience), CD4-biotin (clone RM 4-5, Biolegend), all diluted 1:100 in PBS. Streptavidin-Alexa 546 (1:200 in PBS) was used as secondary antibody for 30 minutes. Nuclei were stained with Hoechst for 3 minutes, diluted 1:1,000 in PBS. Washing steps after antibody incubation and Hoechst staining were performed in 1 × PBS (three times, 2 minutes each). Fluorescence microscopic evaluation was performed on a Carl Zeiss AxioImager Z1. For immunohistochemistry, air-dried slides were incubated with 1% H_2_O_2_ for 10 minutes before blocking with avidin-biotin (Avidin/Biotin Blocking Kit, Vector, Linaris GmBH, Wertheim, Germany) for 15 minutes each, followed by a 15-minute block with 1% FCS. CD90.1-biotin antibody conjugate (clone HIS51) was used for donor T cell staining for 1 h followed by the ABC method (Vector) according to the manufacturer’s protocol.

### Histologic evaluation

For histological analysis organs were fixed in PBS containing 4% paraformaldehyde (PFA) (Roth, Karlsruhe, Germany). Representative PFA fixed samples of GIT, liver and skin of each group were embedded in paraffin, cut into 3-μm thick sections, and stained with hematoxylin and eosin (H&E). GVHD scoring was performed according to Lerner *et al*. [11] based on tissue damage. The scoring system categorized 0 as normal, 1 for mild, 2 for moderate, and 3 for severe tissue damage caused by donor T cells. All slides were examined by an experienced unbiased pathologist (SS).

### Statistical analyses

Statistical analysis was performed with the non-parametric Mann-Whitney test using GraphPad InStat 3 software (GraphPad, La Jolla, CA, USA) where appropriate. Measurements are expressed as the mean ± standard error of mean (SEM). Analysis was performed using GraphPad Prism 5 software. Data reaching statistical significance (*P* ≤0.05) is indicated with an asterisk (*).

## Results and discussion

**Figure 1:**
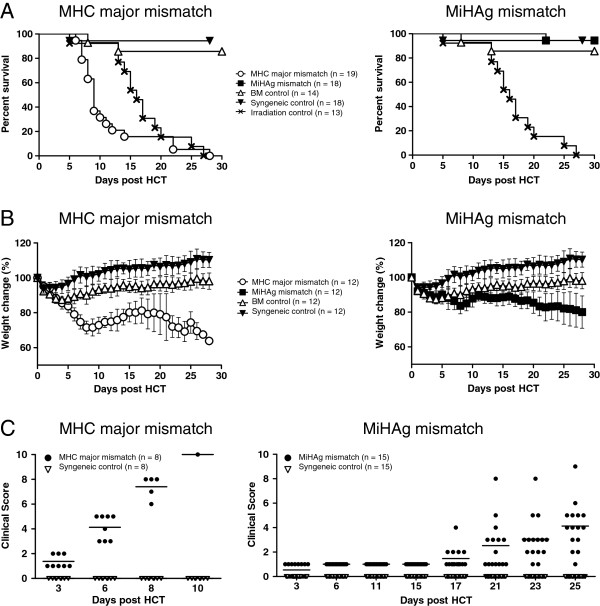
**Differences in disease onset, survival, and clinical score between the acute minor histocompatibility antigen (miHAg) mismatched and the hyperacute major histocompatibility complex (MHC) fully mismatched graft-versus-host disease (GVHD) models.** Myeloablatively conditioned recipients were transplanted as described or left untreated and monitored for weight change, clinical GVHD symptoms, and survival daily after hematopoietic cell transplantation (HCT). **(A)** Weight change is displayed relative to day 0 of HCT. Graphs show summarized data from two independent experiments for MHC major mismatched BALB/c mice (◯, left panel) and miHAg mismatch BALB/b recipients (■, right panel) separately. In both panels a syngeneic control (▼) and a bone marrow control (△) were added. Error bars display means plus or minus SEM. **(B)** Clinical GVHD scoring rapidly increased in symptoms in the MHC major mismatch model (●, left panel). In the miHAg mismatch group (●, right panel) symptoms continuously increased compared to syngeneic controls (▽). (**C**) MHC major mismatch recipients (◯, left panel) developed hyperacute GVHD and most animals died within 14 days after HCT. In contrast, BALB/b recipients (miHAg mismatch, ■, right panel) mostly survived until day +30. Mice that received lethal irradiation without subsequent HCT died from the consequences of myeloablation (×). In both panels a syngeneic control (▼) and a bone marrow control (△) were added. Graphs show summarized data from two independent experiments for MHC major mismatch recipients (◯, left panel) and miHAg mismatch recipients (■, right panel) separately.

Understanding the precise timing of T cell engraftment and cell migration patterns is necessary to further investigate the phenotypic and functional changes within the lymphocyte population in order to develop a predictive test to identify patients at high risk for aGVHD. To study the dynamic pathophysiologic events leading to the initiation of aGVHD we first compared survival, weight change, and clinical aGVHD scoring in a clinically relevant model of miHAg mismatch allo-HCT (B6, H-2^b^ > BALB/b, H-2^b^) to a well-defined MHC major mismatched aGVHD model (B6, H-2^b^ > BALB/c, H-2^d^) [5]. Initially, all miHAg mismatched recipients lost and regained weight similar to syngeneic control mice (Figure 1A) and did not display any clinical signs of aGVHD within the first 2 weeks after allo-HCT (Figure 1B). By day +15, miHAg mismatched BALB/b recipients started to lose weight again and by day +21 mice presented the first signs of diarrhea followed by ruffled fur and hunchback. However, most animals survived until the end of the experiments at day +30 (Figure 1C). All MHC major mismatched BALB/c recipients developed signs of a hyperacute form of GVHD (weight loss, ruffled fur, diarrhea, hunched posture, and lethargy) and most animals succumbed between day +6 and day +14 after HCT (Figure 1A). Transfer of BM cells alone rescued all recipients from myeloablative irradiation except for one BM control mouse that likely did not engraft (Figure 1C). Syngeneic albino B6 recipients (H-2^b^) did not develop any signs of aGVHD (Figure 1B), regained their body weight, and survived until the end of the experiments (Figure 1A,C). Daily *in vivo* BLI measurements confirmed the same spatiotemporal shift of aGVHD initiation to effector phase in both allogeneic models but a diverging signal increase after miHAg mismatch allo-HCT. These early events in aGVHD initiation are in accordance with a seminal study by Korngold and colleges [6]. In contrast, in syngeneic HCT recipients, homeostatic T cell proliferation led to a moderate whole body as well as single organ signal increase compared to allogeneic recipients (see Additional file 1A,B).

**Figure 2:**
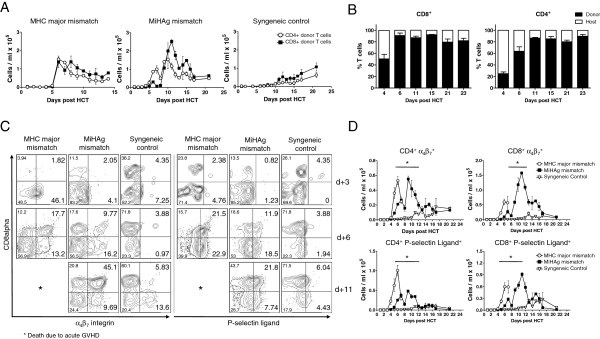
**Migrating donor T cells after minor histocompatibility antigen (miHAg) mismatch allogeneic hematopoietic cell transplantation (allo-HCT) can be clearly detected in the peripheral blood (PB) for at least 2 weeks before onset of acute graft-versus-host disease (aGVHD). (A)** Absolute PB T cell counts for donor CD4^+^ (◯) and CD8^+^ (■) T cells after major histocompatibility complex (MHC) mismatch (left panel), miHAg mismatch (middle panel), and syngeneic HCT (right panel) are shown. Three mice per day per group were analyzed (n = 15 per group). In the MHC major mismatch model, only surviving animals could be bled at later timepoints. One experiment out of three is shown. Error bars display means plus or minus SEM. **(B)** T cell chimerism analysis on indicated timepoints after miHAg mismatch allo-HCT revealed that after day +6 more than 90% of PB CD8^+^ and CD4^+^ T cells were of donor T cell origin. Of note, the detection of CD90.1^+^ T cells only included transferred donor T cells but not donor bone marrow (BM)-derived CD90.2^+^ T cells. Results for CD8^+^ and CD4^+^ T cells of one representative experiment are shown, separately (n = 3). **(C)** Representative flow cytometry plots display α4β7 integrin and P-selectin ligand expression on donor T cells at indicated timepoints in allogeneic and syngeneic recipients. Numbers in each quadrant represent percentages of all donor T cells. **(D)** Daily flow cytometry analyses revealed high numbers of cells in the PB expressing α4β7 integrin as well as P-selectin ligand in the miHAg model (■) in comparison to the expression in syngeneic recipients (▽) which stayed constantly low. Donor T cells in the MHC major mismatch model (◯) also strongly upregulated those receptors before mice died of aGVHD. Three mice per day per group were analyzed (n = 15). Graphs show results from one representative experiment out of three. Error bars display means plus or minus SEM. *P ≤0.05.

Next, we closely monitored donor T cell migration patterns in the clinically relevant miHAg mismatch model to address whether it is possible to detect alloreactive donor T cells in the PB early after allo-HCT. Therefore, we analyzed PB samples of transplanted mice daily by multiparameter flow cytometry (Figure 2A). By employing the well-established MHC major mismatch model of hyperacute GVHD we detected donor T cell mobilization from SLOs to the PB as early as day +4 after allo-HCT. Donor CD4^+^ and CD8^+^ T cell frequencies peaked between day +6 and +8 and decreased until mice succumbed to aGVHD (Figure 2A, left panel). Similarly, after miHAg allo-HCT, donor T cells first mobilized to the PB by day +4, but in contrast to the hyperacute model, elevated numbers of migrating donor CD4^+^ and CD8^+^ T cells remained readily detectable throughout a period of 12 days before cell numbers declined and mice started to show first signs of aGVHD (by day +21) (Figure 2A, middle panel). T cell chimerism analysis after miHAg mismatch allo-HCT revealed that after day +6 more than 90% of PB CD8^+^ and CD4^+^ T cells were of donor T cell origin (Figure 2B). Of note, the detection of CD90.1^+^ T cells only included transferred donor T cells but not donor bone marrow derived CD90.2^+^ T cells. This explains why after day +15 CD90.1^+^ donor T cells apparently decline. However, this cannot be ascribed to an increase of host type T cells but rather to an increase of donor bone marrow CD90.2^+^ T cells that could not be detected by our type of chimerism analysis (Figure 2B). In syngeneic recipients donor T cell numbers slowly but constantly increased suggesting homeostatic T cell reconstitution of the immune system (Figure 2A, right panel). Histopathological analyses of aGVHD target organs confirmed the donor T cell infiltration in these organs. By day +6 after MHC major mismatch allo-HCT allogeneic T cells markedly infiltrated target organs resulting in massive tissue damage. In contrast, miHAg mismatch allo-HCT recipients displayed low intestinal T cell infiltration and only mild histopathological damage on day +6. However, by day +21, donor T cells massively infiltrated small and large bowel of miHAg mismatch allo-HCT recipients that by this time displayed the first clinical signs of aGVHD. The tissues of syngeneic controls displayed only mild T cell infiltrates and no tissue damage at this time (see Additional file 2A,B). As aGVHD target organ infiltration depends on the appropriate cellular homing receptors [3] we considered whether alloreactive T cells could be more precisely characterized by the expression of the homing receptors α4β7 integrin and P-selectin ligand, implicated in intestinal and skin aGVHD manifestation, respectively [12,13]. Therefore, we examined the dynamic changes in their expression daily after HCT to test a possible correlation between their upregulation and the identified phase of massive cell migration (Figure 2C,D). Until day +3, only few donor T cells circulated in the PB in all three models and these cells were mostly negative for the receptors tested. On day +6, donor T cells of the MHC major mismatch model upregulated α4β7 integrin and P-selectin ligand compared to a minor upregulation of those receptors after miHAg allo-HCT. Importantly, in the latter model upregulation of α4β7 integrin and P-selectin ligand peaked at day +11 (45% α4β7^+^ CD8^+^ and 22% P-lig^+^ CD8^+^ donor T cells, Figure 2D). After day +11, numbers of cells expressing these homing receptors slowly declined correlating with a drop in absolute cell numbers. In contrast, donor T cells in syngeneic transplanted mice expressed only low levels of α4β7 integrin and P-selectin ligand over time.

In line with our results, two clinical retrospective studies indicate a correlation between homing receptor upregulation and the subsequent development of aGVHD symptoms. Chen and colleagues found at least tenfold upregulation of α4β7 integrin on naïve as well as memory T cell subsets before the onset of clinically apparent intestinal GVHD compared to cutaneous or no aGVHD in a case-controlled study including 59 patients [14]. Another study with 33 patients found an association of cutaneous lymphocyte antigen (CLA, a P-selectin glycoprotein ligand-1)-upregulation on PB T cells and the onset of severe aGVHD [15]. Furthermore, in a recent clinical study Chen *et al*. could correlate the detection of α4β7^+^ memory T cells with the appearance of intestinal aGVHD in patients [16]. However, these studies did not address how soon before aGVHD onset and for how long these T cell surface receptors could be detected.

Different functional studies in murine models across major or minor MHC mismatches confirmed that α4β7 or ß7 negative donor T cells cause less aGVHD morbidity and mortality compared to WT T cells mainly due to reduced homing to the intestinal tract while still exhibiting the GVT effect [12,17]. Likewise, ameliorated aGVHD was observed in P-selectin-deficient mice, which underscores the importance of the interaction between P-selectin and its ligands that are highly expressed on migrating effector T cells [13]. In our experiments we also found significantly higher α4β7 integrin and P-selectin ligand expression on migrating allogeneic T cells than in syngeneic controls. The expression of these molecules on PB donor T cells correlates with the identified cell migration phase and strongly associates with aGVHD induction in allo-HCT suggesting their potential usefulness as predictive markers. Recently, research efforts have focused on the identification of reliable predictive markers to identify patients at risk before aGVHD onset with some potential biomarkers being investigated thoroughly [18,19]. Different combinations of these biomarkers have been used to predict treatment response and survival of aGVHD patients, but a predictive marker panel to reliably identify patients at risk for aGVHD is still lacking.

**Figure 3:**
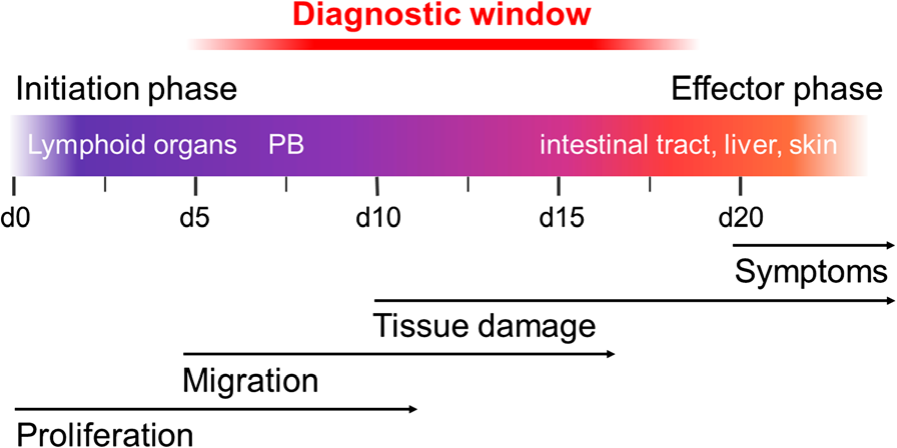
**Schematic summary of events in the minor histocompatibility antigen (miHAg) mismatch model early after allogeneic hematopoietic cell transplantation (allo-HCT).** Donor T cell migration patterns open an at least 2-week-long diagnostic window prior to clinical apparent acute graft-versus-host disease (aGVHD) onset. Color bar displays days after allo-HCT. PB, peripheral blood.

Figure 3 summarizes the dynamic events of aGVHD pathophysiology after miHAg mismatch allo-HCT. The described donor T cell migration phase with the cells being highly positive for α4β7 integrin and P-selectin ligand opens a potential diagnostic window for a timely therapeutic intervention prior to the onset of clinically apparent aGVHD, which did not develop before day +21 in this model.

**Figure 4:**
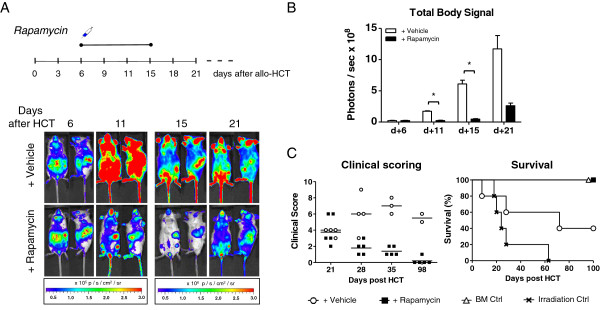
**Targeted rapamycin treatment starting at the first detection of peripheral blood (PB) alloreactive T cells prevents lethal acute graft-versus-host disease (aGVHD). (A)** Treatment was started as soon as alloreactive T cells could be identified in the PB based on their surface receptor profile. Scheme indicates time course of repeated rapamycin injections. Ventral and lateral bioluminescence imaging (BLI) images of representative mice per group (n = 5) are displayed. On days +15 and +21, signal thresholds were increased to resolve the predominant organ distribution of donor T cells. **(B)** BLI signals significantly decreased on days +11 and +15 in treated mice compared to vehicle controls (n = 5). Error bars display means plus or minus SEM. *P ≤0.05. **(C)** All rapamycin-treated mice (■) showed limited signs of aGVHD (left panel) on day +21 but rapidly and fully recovered. All rapamycin-treated mice survived (right panel) until the end of the experiment (day +100). In contrast, vehicle controls (◯) developed increasing signs of aGVHD starting by day +21 and 60% succumbed to aGVHD up to day +100. Mice that received lethal irradiation without subsequent hematopoietic cell transplantation (HCT) died of the consequences of myeloablation (×). Animals that received transplants of bone marrow alone did not show any clinical signs of aGVHD and survived (△). One experiment out of two is shown (n = 5).

Therefore, we asked whether this time frame from the first detection of alloreactive T cells suffices to effectively prevent aGVHD onset. Subsequently, we started to treat mice with rapamycin [20,21] as soon as we could detect alloreactive T cells in the PB (day +6) (Figure 4A). A total of 10 days of rapamycin administration strongly reduced the total body BLI signal intensity of treated mice compared with vehicle controls. By day +11, treated mice already showed much less overall body signal predominantly originating from the spleen, skin, and BM compartment. Abdominal signals were very low compared with the controls. Immunofluorescence stainings of donor T cell infiltrates in aGVHD target organs confirmed reduced CD90.1^+^ donor T cells in rapamycin-treated mice compared to controls on day +11 after miHAg mismatch allo-HCT (see Additional file 3A-D). Quantification of BLI images revealed a significant reduction of total body signal intensities by day +11 and day +15 after allo-HCT (Figure 4B). Treated mice showed a 7-fold lower signal by day +11 and a 13-fold lower signal by day +15 compared with vehicle controls. By the end of treatment (day +15) the signal from the treated group slightly increased but the benefit of the treatment remained apparent with a fourfold lower BLI signal compared to control mice. Importantly, rapamycin treatment resulted in the recovery from already visible aGVHD symptoms occurring by day +21 (Figure 4C) that constantly improved thereafter. All mice treated in a timely fashion with rapamycin survived (Figure 4C).

We propose that the identified migration phase of alloreactive T cells may serve as a potential diagnostic window for early and timely targeted therapeutic interventions. In addition to the described surface markers, α4β7 integrin and P-selectin ligand, combinations with other markers appear attractive. Further investigations of suitable surface receptor combinations to precisely identify alloreactive PB T cells could be complemented by detecting polypeptides in the urine of patients as early indicators for aGVHD. Kaiser *et al*. identified two peptides of the leukotriene A4 hydrolase and of serum albumin as possible biomarkers [22]. Furthermore, tumor necrosis factor receptor 1 (TNFR1), interleukin 2 receptor alpha (IL-2Ra), IL-8, and hepatocyte growth factor (HGF) as plasma biomarkers relevant for aGVHD effectively discriminated between patients with and without aGVHD [23] and elafin functioned as a biomarker for skin aGVHD patients [24]. Different combinations of these biomarkers have been used to predict treatment response and survival of aGVHD patients [18,19]. We envision combining the described homing receptor panel with these biomarkers in order to develop a reliable predictive test to identify patients at risk before disease onset.

Encouraging for the clinical allo-HCT setting, the directed, preemptive rapamycin treatment starting immediately at the first detection of alloreactive donor T cells significantly improved aGVHD symptoms and granted survival in all miHAg allo-HCT recipients. Similarly, preemptive treatment with other immunomodulators and, particularly, drugs altering T cell trafficking [25] appear highly attractive, but may exert their biggest benefit if individuals at high risk for aGVHD can be identified in a timely manner before the first clinical symptoms appear.

## Conclusions

In the present study, we demonstrate in a clinically relevant miHAg allo-HCT mouse model that it is feasible to detect migrating alloreactive donor T cells for an extended time period of 2 weeks and, importantly, before aGVHD symptoms become apparent. As the prediction of aGVHD is highly relevant to the clinical outcome, it may be beneficial to closely monitor the kinetics of T cell engraftment, expansion, and homing receptor expression in allo-HCT patients. Our preclinical findings may have implications for the development of a predictive blood test identifying patients at risk for aGVHD and thereby giving physicians the chance for a timely and targeted therapeutic intervention.

## Competing interests

The authors declare they have no competing interests.

## Authors’ contributions

CAB, SSR, JB performed and CB, A-LJG, MC, MR, RZ helped with experiments, CAB, SS, GFB and AB analyzed data. SS performed histopathological scoring. GFB performed statistical analyses. MC, SSR, GFB, RZ, PGS, RSN and HE helped write the paper. CAB and AB designed research and wrote the paper. All authors read and approved the final manuscript.

## Pre-publication history

The pre-publication history for this paper can be accessed here:

http://www.biomedcentral.com/1741-7015/11/134/prepub

## Supplementary Material

Additional file 1 **Initial donor T cell proliferation and migration after allogeneic hematopoietic cell transplantation (allo-HCT) follows the same spatiotemporal pattern but differs in bioluminescence imaging (BLI) signal increase in the major histocompatibility complex (MHC) versus the minor histocompatibility antigen (miHAg) mismatched mouse model. (A)***Luc*^+^ donor T cells proliferate in secondary lymphoid organs (SLOs) until day +3 in both allogeneic models. They show the same spatiotemporal shift from acute graft-versus-host disease (aGVHD) initiation to effector phase but in the miHAg mismatch model (middle panel) signals increase less intensely compared to the MHC major mismatch model (upper panel) during the first 7 days followed by a strong increase in whole body signal intensity from day +8 on. In syngeneic transplanted albino B6 mice (lower panel) donor T cells initially also home to SLOs but as well to the thymus and bone marrow (BM) and not to the skin. One representative mouse per group (n = 6) is shown. **(B)** Quantification of dynamic total body and single organ BLI signal changes. Photon emissions per second per whole animal and average photon radiation for representative target organs are displayed. Total body BLI signals increase dramatically in mice with aGVHD in both allogeneic models, MHC major (●) and miHAg (■) mismatched groups, but only moderately in syngeneic (▼) transplanted mice. Overall gastrointestinal tract (GIT) and skin signal intensities of the MHC major mismatched mice increase much stronger than in the miHAg mismatched model before those mice die of aGVHD. Error bars display means plus or minus SEM (n = 6). Click here for file

Additional file 2 **Alloreactive donor T cell infiltrates cause severe tissue damage in acute graft-versus-host disease (aGVHD) target organs.** Immunohistochemical stainings of representative small **(A)** and large **(B)** bowel samples on day +6 after hematopoietic cell transplantation (HCT) showed a massive infiltration by donor T cells in the major histocompatibility complex (MHC) mismatch model as compared to minor histocompatibility antigen (miHAg) mismatch and syngeneic recipients’ tissues. By day +21, donor T cells infiltrated small and large bowel of the miHAg mismatch transplanted mice and were more abundant than in the organs of syngeneic recipients. Immunofluorescence microscopy of target tissues on day +21 after HCT in the miHAg mismatched compared to syngeneic recipients confirmed these results ((A) and (B), lower panels). CD4^+^ (Streptavidin-Alexa546, displayed in blue) as well as CD8^+^ (Alexa-488, displayed in green) donor T cells (CD90.1-APC, displayed in red) massively infiltrated small and large bowel of miHAg mismatch recipients. Double-positive cells appear in purple (CD90.1^+^ CD4^+^) and yellow (CD90.1^+^ CD8^+^), respectively. In syngeneic recipients, double-positive donor T cells also migrated into these organs, but unlike in allogeneic recipients did not cause tissue damage. **(C)** Quantification of infiltrating donor T cells showed that more cells infiltrated the small and large bowel in allogeneic than in syngeneic recipients on day +6 (left panel) as well as on day +21 (right panel) after allo-HCT. Similar results were seen in liver and skin. One of three independent experiments is shown (n = 3). Error bars display means plus or minus SEM. HPF, high power field. Click here for file

Additional file 3 **There is less donor T cell infiltration in acute graft-versus-host disease (aGVHD) target organs in rapamycin-treated mice than in aGVHD control animals.** On day +11 after minor histocompatibility antigen (miHAg) mismatch allogeneic hematopoietic cell transplantation (allo-HCT), donor T cells (CD90.1-APC, displayed in green) massively infiltrated small **(A)** and large bowel **(B)** in aGVHD and vehicle control animals whereas the gastrointestinal tract of rapamycin-treated mice showed less donor cell infiltration. Similar results were seen in liver **(C)** and skin **(D)** samples. Images show immunofluorescence stainings of one representative mouse per group (n = 5). Cell nuclei were stained with 4′,6-diamidino-2-phenylindole (DAPI) (displayed in white). Images were taken with 40 × magnification. LB, large bowel; SB, small bowel. Click here for file
