# Supplementary material for: A diagnostic window for the treatment of acute graft-versus-host disease prior to visible clinical symptoms in a murine model
Source: BMC Med. 2013 May 21;11:134. doi: 10.1186/1741-7015-11-134 (PMC3665617; doi:10.1186/1741-7015-11-134)
Supplement: Additional file 1 — Initial donor T cell proliferation and migration after allogeneic hematopoietic cell transplantation (allo-HCT) follows the same spatiotemporal pattern but differs in bioluminescence imaging (BLI) signal increase in the major histocompatibility complex (MHC) versus the minor histocompatibility antigen (miHAg) mismatched mouse model. (A)Luc+ donor T cells proliferate in secondary lymphoid organs (SLOs) until day +3 in both allogeneic models. They show the same spatiotemporal shift from acute graft-versus-host disease (aGVHD) initiation to effector phase but in the miHAg mismatch model (middle panel) signals increase less intensely compared to the MHC major mismatch model (upper panel) during the first 7 days followed by a strong increase in whole body signal intensity from day +8 on. In syngeneic transplanted albino B6 mice (lower panel) donor T cells initially also home to SLOs but as well to the thymus and bone marrow (BM) and not to the skin. One representative mouse per group (n = 6) is shown. (B) Quantification of dynamic total body and single organ BLI signal changes. Photon emissions per second per whole animal and average photon radiation for representative target organs are displayed. Total body BLI signals increase dramatically in mice with aGVHD in both allogeneic models, MHC major (●) and miHAg (■) mismatched groups, but only moderately in syngeneic (▼) transplanted mice. Overall gastrointestinal tract (GIT) and skin signal intensities of the MHC major mismatched mice increase much stronger than in the miHAg mismatched model before those mice die of aGVHD. Error bars display means plus or minus SEM (n = 6). [file 1741-7015-11-134-S1.pptx]

## Slide 1
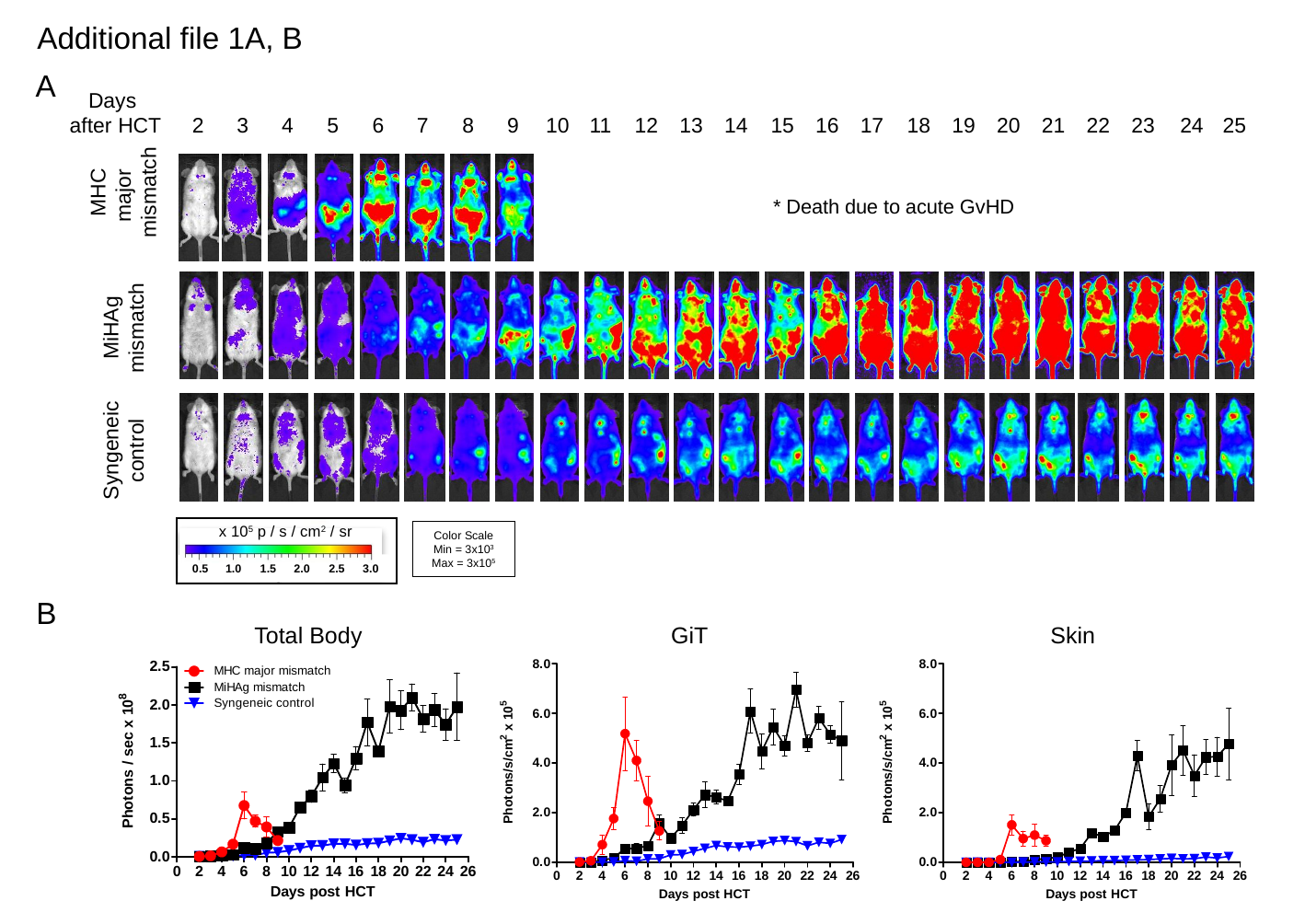

Additional file 1A, B
A
Days
after HCT
2
3
4
5
6
7
8
9
10
11
12
13
14
15
16
17
18
19
20
21
22
23
24
25
MHC
major
mismatch
MiHAg
mismatch
Syngeneic
control
x 105 p / s / cm2 / sr
3.0
0.5
1.0
1.5
2.0
2.5
Color Scale
Min = 3x10^3^
Max = 3x10^5^
* Death due to acute GvHD
B
Total Body
GiT
Skin
