# Supplementary material for: A diagnostic window for the treatment of acute graft-versus-host disease prior to visible clinical symptoms in a murine model
Source: BMC Med. 2013 May 21;11:134. doi: 10.1186/1741-7015-11-134 (PMC3665617; doi:10.1186/1741-7015-11-134)
Supplement: Additional file 2 — Alloreactive donor T cell infiltrates cause severe tissue damage in acute graft-versus-host disease (aGVHD) target organs. Immunohistochemical stainings of representative small (A) and large (B) bowel samples on day +6 after hematopoietic cell transplantation (HCT) showed a massive infiltration by donor T cells in the major histocompatibility complex (MHC) mismatch model as compared to minor histocompatibility antigen (miHAg) mismatch and syngeneic recipients’ tissues. By day +21, donor T cells infiltrated small and large bowel of the miHAg mismatch transplanted mice and were more abundant than in the organs of syngeneic recipients. Immunofluorescence microscopy of target tissues on day +21 after HCT in the miHAg mismatched compared to syngeneic recipients confirmed these results ((A) and (B), lower panels). CD4+ (Streptavidin-Alexa546, displayed in blue) as well as CD8+ (Alexa-488, displayed in green) donor T cells (CD90.1-APC, displayed in red) massively infiltrated small and large bowel of miHAg mismatch recipients. Double-positive cells appear in purple (CD90.1+ CD4+) and yellow (CD90.1+ CD8+), respectively. In syngeneic recipients, double-positive donor T cells also migrated into these organs, but unlike in allogeneic recipients did not cause tissue damage. (C) Quantification of infiltrating donor T cells showed that more cells infiltrated the small and large bowel in allogeneic than in syngeneic recipients on day +6 (left panel) as well as on day +21 (right panel) after allo-HCT. Similar results were seen in liver and skin. One of three independent experiments is shown (n = 3). Error bars display means plus or minus SEM. HPF, high power field. [file 1741-7015-11-134-S2.pptx]

## Slide 1
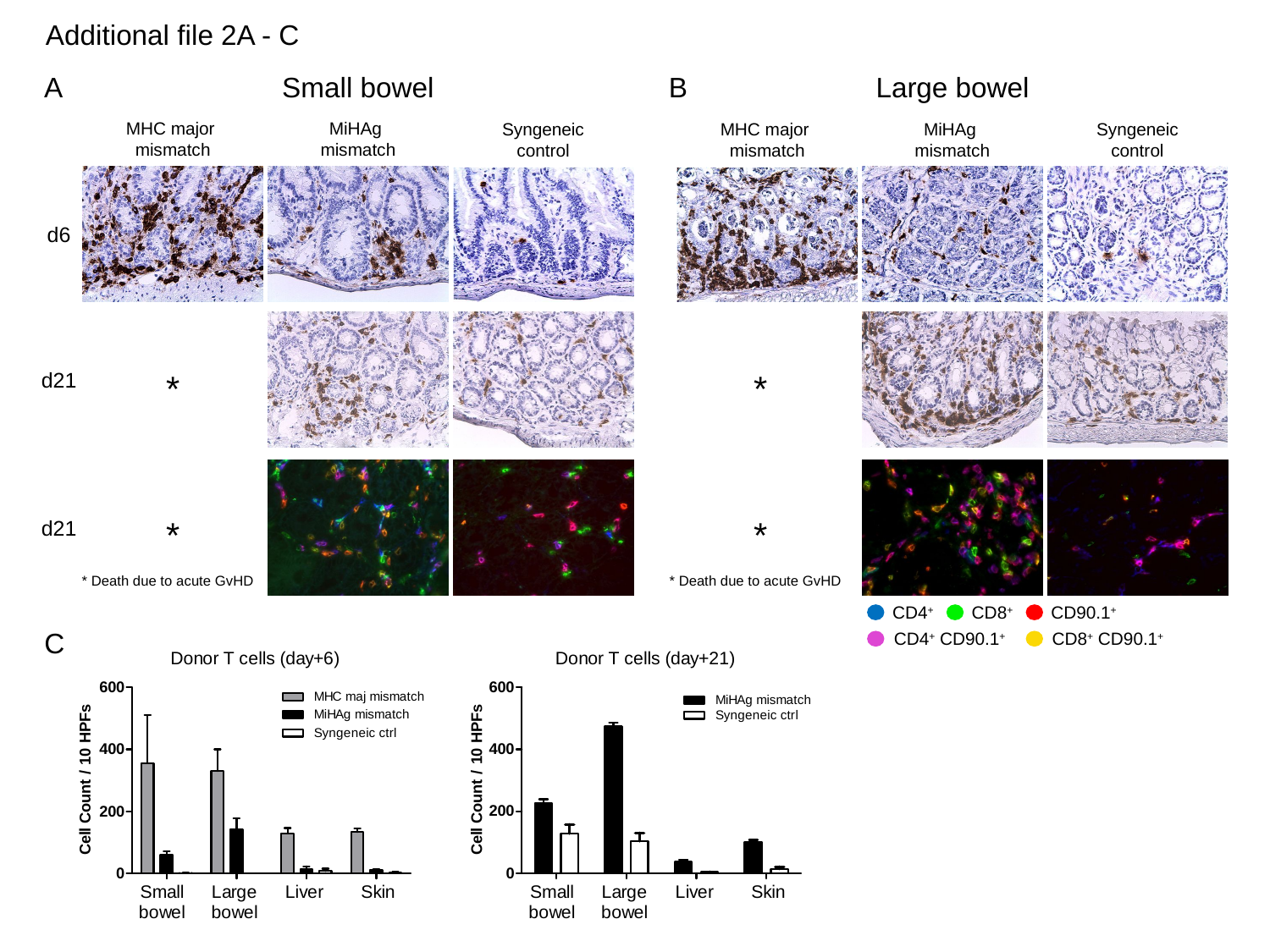

Additional file 2A - C
A
Small bowel
B
Large bowel
MHC major
mismatch
MiHAg
mismatch
Syngeneic
control
MHC major
mismatch
MiHAg
mismatch
Syngeneic
control
d6
d21
d21
CD4+
CD8+
CD90.1+
CD4+ CD90.1+
CD8+ CD90.1+
*
*
*
*
* Death due to acute GvHD
* Death due to acute GvHD
C
