# Supplementary material for: A diagnostic window for the treatment of acute graft-versus-host disease prior to visible clinical symptoms in a murine model
Source: BMC Med. 2013 May 21;11:134. doi: 10.1186/1741-7015-11-134 (PMC3665617; doi:10.1186/1741-7015-11-134)
Supplement: Additional file 3 — There is less donor T cell infiltration in acute graft-versus-host disease (aGVHD) target organs in rapamycin-treated mice than in aGVHD control animals. On day +11 after minor histocompatibility antigen (miHAg) mismatch allogeneic hematopoietic cell transplantation (allo-HCT), donor T cells (CD90.1-APC, displayed in green) massively infiltrated small (A) and large bowel (B) in aGVHD and vehicle control animals whereas the gastrointestinal tract of rapamycin-treated mice showed less donor cell infiltration. Similar results were seen in liver (C) and skin (D) samples. Images show immunofluorescence stainings of one representative mouse per group (n = 5). Cell nuclei were stained with 4′,6-diamidino-2-phenylindole (DAPI) (displayed in white). Images were taken with 40 × magnification. LB, large bowel; SB, small bowel. [file 1741-7015-11-134-S3.pptx]

## Slide 1
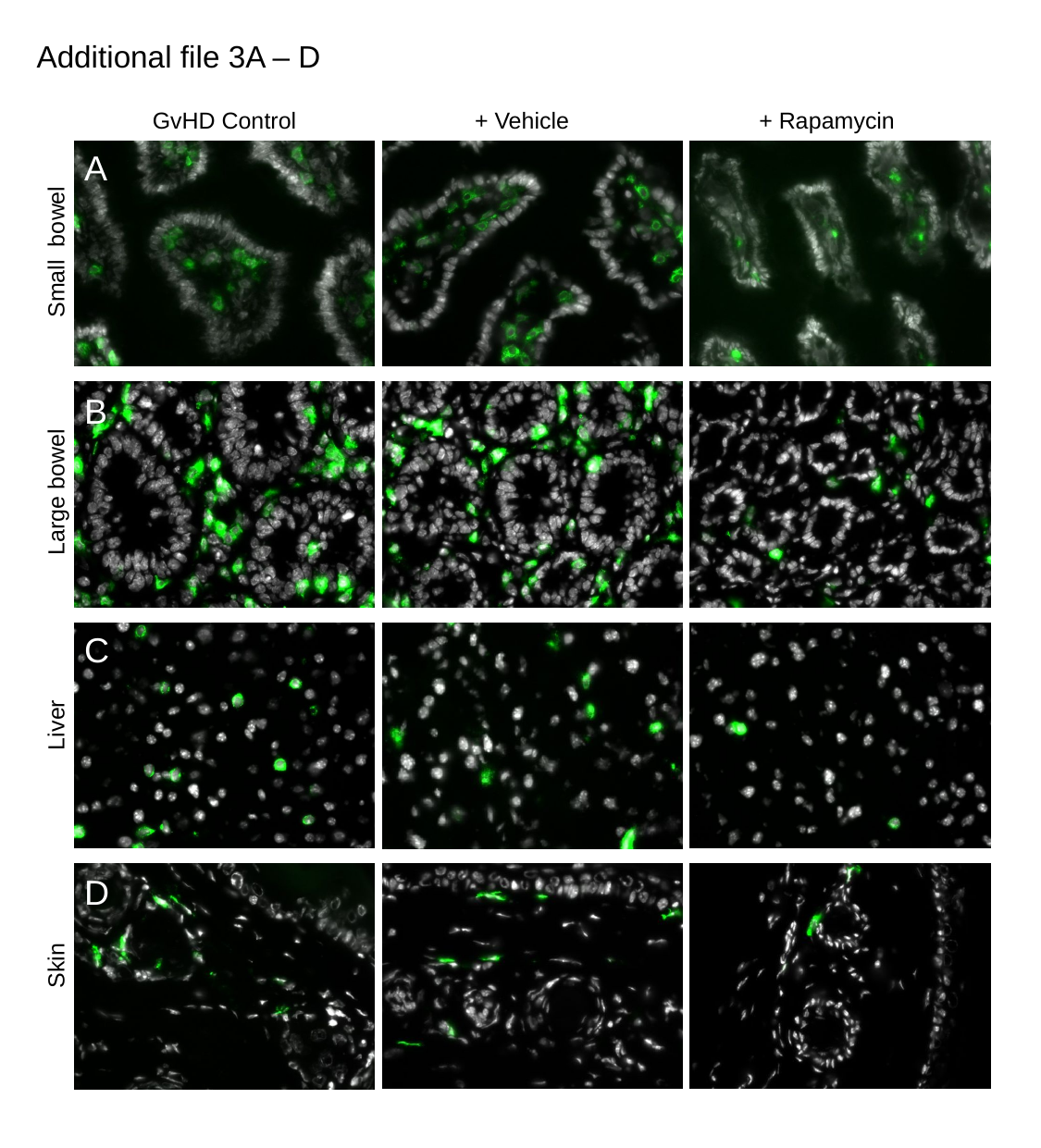

Additional file 3A – D
GvHD Control
+ Vehicle
+ Rapamycin
A
Small bowel
B
Large bowel
C
Liver
D
Skin
